# Slow pyrolysis of *Terminalia catappa* L. municipal solid waste and the use of the aqueous fraction produced for bovine mastitis control

**DOI:** 10.1016/j.bbrep.2024.101704

**Published:** 2024-04-09

**Authors:** Rafaelle Vinturelle, Taissa da Silva Cabral, Pamella C.O. de Oliveira, Juliana P. Salles, Juliana V. Faria, Guilherme P. Teixeira, Robson X. Faria, Márcia C.C. Veloso, Gilberto A. Romeiro, EvelizeFolly das Chagas

**Affiliations:** a Laboratory of Pest and Parasite Studies – Federal Fluminense University–Institute of Biology– Department of Cellular and Molecular Biology – Niterói, RJ, Brazil, CEP 24210-201; b Postgraduate Program in Chemistry – Federal Fluminense University – Niterói, RJ, CEP; 24.020-141, Brazil; c Postgraduate Program in Science and Biotechnology – Federal Fluminense University – Niterói, RJ, CEP: 24.210-201, Brazil; d Laboratory for Environmental Health Assessment and Promotion, Oswaldo Cruz Institute, Manguinhos, Rio de Janeiro, RJ, 21040-900, Brazil; e National Institute of Sciences and Technology - Molecular Entomology INCT-EM – Brazil, Rio de Janeiro, Brazil; f Laboratory of Studies in Experimental Pharmacology, Federal University of Rio de Janeiro, Ilha do Fundão, Rio de Janeiro, RJ, 21941-590, Brazil; g Laboratory of Synthesis, Chromatography and Environment (SINCROMA) – Federal Fluminense University – Institute of Chemistry – Department of Organic Chemistry – Niterói, RJ, Brazil

**Keywords:** *Terminalia catappa* L., Pyrolysis, Aqueous fraction, Bovine mastitis

## Abstract

The *Terminalia catappa* L. tree is an ornamental and shade tree producter of a large amount of biological waste sent to landfills. Therefore, this plant constitutes so-called municipal solid wood waste (MSWW), which causes undesirable impacts on the environment, such as the generation of methane through the action of microorganisms. Sustainable solutions for the proper use and disposal of MSWW are a topic that has assumed great relevance at present due to the high quantities of MSWW generated worldwide. Pyrolysis constitutes an attractive alternative for the sustainable use of MSWW to produce higher value-added products. This study investigated the slow pyrolysis of *Terminalia catappa* L. fruit and the use of the aqueous fraction produced for bovine mastitis control. We obtained four fractions from the pyrolysis process, with average yields of the aqueous phase (36.22 ± 2.0 %), bio-oil (5.52 ± 0.4 %), biochar (37.55 ± 2.8 %) and gas (20.71 ± 2.0 %). The aqueous fraction was extracted with organic solvents and analyzed by gas chromatography coupled to mass spectrometry (GC‒MS). The extracts were composed mainly of phenols (50 %), furan derivatives, cyclic ketones, and others with lower contents, such as alcohols and esters. The aqueous fraction had bactericidal activity against *Staphylococcus aureus, Klebsiella pneumoniae, Pseudomonas aeruginosa and Escherichia coli,* which are responsible for bovine mastitis. In addition, the fraction showed low cytotoxicity against a murine melanoma cell line from a C57BL/6J mouse, B16F10 cells and mouse peritoneal cells.

## 1 Introduction

**The***Terminalia catappa* L. (*T. catappa*) tree of the Combretaceae family, popularly known as tropical, ketapang, beach or Indian almond, grows well in subtropical and tropical zones. This plant originated in a region known as South Asia. However, human migration has forced extensive cultivation of this species as an ornamental and shade tree in several tropical countries. In Brazil, this plant can be found close to the coastal environment [1,2]. Interestingly, the seeds of the ketapang fruit are composed of vegetable oil and biodiesel, in addition to having promising physicochemical properties, which makes this plant suitable for industrial use [3]. *T. catappa* phytochemically contains a variety of compounds, such as phenols, tannins, and flavors. Tannins and phenolic compounds are mainly responsible for the biological activity of plants, including their antimicrobial activity [4,5].

**Fig. 1:**
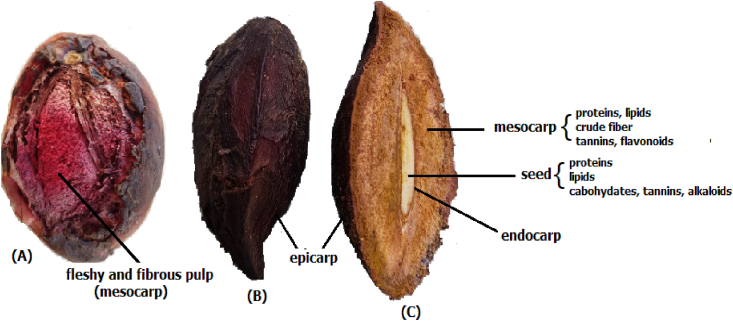
*T*. *catappa* fruit and its components: (A) ripe fruit; (B) ellipsoid fibrous drupe; (C) cross section.

In Brazil, commonly called almond or castanets, *T. catappa* is a fibrous ellipsoid drupe with thin skin (epicarp) and a mesocarp with a fleshy, fibrous edible pulp and a hard endocarp containing an edible seed inside (Fig. 1) [1,6]. During ripening, the color changes from green to yellow and dark to purplish red. The chemical composition of the pulp includes lipids, proteins, crude fiber, minerals, tannins, flavonoids, and triterpenes [2,7]. The seed is not exploited commercially. Their approximate analysis revealed a high content of proteins and lipids (whose composition is predominantly oleic (C18:1) and linoleic (C18:2) unsaturated acids, in addition to carbohydrates and minerals [4,6,[8], [9], [10], [11]]). *C.* aphrodisiac activity and moderate consumption are useful in the treatment of sexual dysfunction among men, especially for premature ejaculation [12]. Recently, an aqueous extract from T. Increased glucose transporter 4 (GLUT 4) translocation and activation of the PI3K/AKT pathway were detected in the leaves of Wistar rats fed a fatty diet, which suggests that these plants are active in processes such as insulin resistance and type 2 diabetes [13]. Likewise, *T. catappa* extract also showed a cardioprotective effect in rats through the modulation of respiratory and lysosomal biomarkers [14].

*T. catappa* has great nutritional potential and is used globally in traditional medicines, mainly in African countries. Interestingly, studies on the commercial application of T have been conducted. Catappa is an antifungal, anti-inflammatory, nephroprotective, and antibacterial agent [2,[15], [16], [17], [18], [19], [20]]. In Brazil, this plant is an underused crop, and it continues to be an ornamental and shade tree. Fruits are rarely consumed or are only consumed by children and some animals who generally consume pulp and sometimes also seeds. Uneaten parts are discarded and fall to the ground along with fruits and leaves. Additionally, although the species is deciduous, it can shed two or more times a year during periods of drought to conserve internal moisture. These organic residues from *T. catappa* trees are found in large quantities and are sent daily to sanitary landfills, thus constituting municipal solid wood residues (MSWWs), which have an undesirable impact on the environment. A great example is the release of methane through the action of microorganisms, a gas that stands out as a mediator of climate change [21].

Sustainable solutions for the correct use and disposal of MSWW are highly relevant due to the large amount of waste generated worldwide. Recycling methods such as composting and anaerobic digestion are widely used as sustainable measures. These methods use bacteria as agents that degrade organic matter, biotransforming it and generating renewable products [22]. However, some limitations accompany its practice, mainly due to the risk of secondary pollution, long treatment period and generation of odor nuisance, which has a significant environmental impact [23,24]. In this context, the pyrolysis technique constitutes a promising alternative for the use of agroforestry, which basically consists of a thermal decomposition process in the absence of oxygen. This process leads to the conversion of biomass into products with higher added value, such as liquid fractions (bio-oil and aqueous phase), biochar and noncondensable gases [[25], [26], [27], [28], [29]].

Our research group has already applied this technique to waste for a variety of fields, such as agricultural, industrial and urban fields [[30], [31], [32]], according to the method developed in the 1980s by Bayer and Kutubuddin [33]. Pyrolysis is realized at approximately 380–400 °C, generating liquid and biochar fractions in place of the gaseous fractions. The pyrolysis studies of Terminalia fruit described in the literature only report the use of the obtained biochar as an adsorbent and do not mention the aqueous phase [34,35]. Pyroligneous acid or wood vinegar is referred to as the aqueous phase of the process. Lignocellulosic biomass, when derived from pyrolysis, has several properties, such as antioxidant, fertilizer, pesticide and antimicrobial properties. This activity is probably due to the high presence of phenols, furan and pyran derivatives, nitrogenous compounds, aldehydes, carboxylic acids, ketones, and esters [[36], [37], [38]]. Recently, new antimicrobial agents obtained from biomass have been gaining ground as new alternative or complementary therapeutic sources for the treatment of various diseases due to their low cost and reduced side effects when associated with synthetic drugs [[39], [40], [41]].

Mastitis is a significant inflammatory disease affecting cows worldwide [42]. The exact cause of this disease is uncertain and may be multifactorial. However, mastitis is usually associated with bacterial infections [43,44]. The significant impact of the infection results in a reduced quality of milk production, which leads to severe economic losses estimated at approximately US$2 billion per year [[45], [46], [47]]. The main bacteria highlighted in mastitis are *Streptococcus agalactiae* (*S. agalactiae*), *Staphylococcus aureus* (*S. aureus*), *Mycoplasma* spp.*, Streptococcus uberis* (*S. uberis*), *Streptococcus dysgalactiae* subsp *dysgalactiae* (SDSD), *Pseudomonas aeruginosa* (*P. aeruginosa*) and coliforms, including *Escherichia coli* (*E. coli*) and *Klebsiella* spp [48,49]. Mastitis can be classified into three phases: clinical, subclinical and chronic mastitis. In the first phase, it is easy to verify the inflammatory process, such as swelling, pain and changes in the aspects of the milk, such as clots, purulent secretion, and blood. Subclinical mastitis does not show clinical signs in animals and is a marked manifestation of changes in milk composition. An increase in the somatic cell count, which represents the number of defense cells of the immune system, is an example. In contrast, chronic mastitis can last for several months, with clinical flare-ups occurring at irregular intervals [[50], [51], [52]]. Inflammatory biomarkers are highly expressed during the disease process, and the interleukin cytokines IL-1 and IL-6, tumor necrosis factor α (TNF-α), serum amyloid A (SAA), and haptoglobin (Hp) are the main biomarkers [53].

The main measure to combat the disease is disinfection and cleaning of the facilities before and after milking. On the other hand, cows that develop mastitis often require pharmacotherapy with antibiotic intervention [47]. Two major problems are related to the route of administration (intramamary) and the growth of resistant bacteria, which further complicate the welfare and treatment of affected animals [49,54]. Another factor is the ease of transfer from administered antibiotics to milk in the mammary gland. Therefore, this is the main cause of the presence of residues [55]. Interestingly, new approaches are being undertaken using protein biosensors to detect pathogens present in samples [[56], [57], [58]]. Based on data on the pharmacological activities of fruit and seed extracts, this study was carried out to evaluate the antibacterial activities of the aqueous fraction obtained during the process of slow pyrolysis of *T. catappa* fruits against bovine mastitis. Thus, this work investigated the pyrolysis of *T. catappa* as a possible potential agent for the control of mastitis through antimicrobial activity.

## 2 Materials and methods

### 2.1 Sample collection

Ripe fruits of *T. catappa* were collected from the ground during the dry season from different trees in a metropolitan area of Rio de Janeiro, Brazil (Barra da Tijuca beach, 22054′ 13″ S and 430 12′ 35″ W). The samples were dried in an oven at 110 °C for 24 h before the pyrolysis process.

### 2.2 Proximate and ultimate analysis of *T*. *catappa* fruits

The moisture, volatile and ash contents were determined according to the ASTM D1762-84 method of the American Society for Testing and Materials (ASTM) [59]. The fixed carbon content was determined following the ASTM D3172 protocol [60].

### 2.3 Pyrolysis experimental procedure

Approximately 300 g of *T. catappa* fruit (mesocarp + endocarp + seed) was used per experiment. Slow pyrolysis was realized in a borosilicate fixed bed horizontal reactor, model Heraeus R/O 100, with a N_2_ atmosphere, a temperature of up to 400 °C, a heating rate of 15 °C min^−1^ and a residence time of 2 h. A schematic of the complete system employed and a more detailed explanation of the experimental procedure can be found elsewhere [30,60]. Volatiles generated during pyrolysis were condensed through a glass condenser using cold water as a cooling medium. The condensed phase, composed of bio-oil and an aqueous phase, was collected, measured and weighed. Each experimental protocol at the same temperature was repeated four times. The product yield was the mean value of the four analyses.

### 2.4 Gas chromatographic and mass spectrometric (GC‒MS) analysis of the aqueous phase

The crude aqueous phase of *T. catappa* fruit pyrolysis was subjected to liquid‒liquid extraction using hexane, dichloromethane and ethyl acetate. The obtained organic extracts were dried with powdered anhydrous magnesium sulfate, concentrated in a rotary evaporator and subjected to analysis by GC‒MS.

### 2.5 GC‒MS

GC‒MS analysis was performed using a Shimadzu QP2010. One microliter of each organic extract was injected onto the GC column. A DB-5MS fused silica capillary column (30 m, 0.25 mm i.d., 1.40 μm film thickness) was used in the analysis. The initial oven temperature was 40 °C (5 min hold) and was increased to 230 °C at 5 °C min-1 (10 min hold). Helium was used as the carrier gas at a flow rate of 0.6 mL min^−1,^ and the injector temperature (split mode) was 280 °C. The mass detector was operated under the following conditions: the temperature was 250 °C, the electron ionization energy was 70 eV, and the MS interface temperature was maintained at 200 °C. The data were collected in full scan mode (range, 40–600 Da), and a 4 min solvent delay was used. OMCs were identified by comparing their mass spectra with the National Institute of Standards and Technology (NIST) 27 and 147 mass spectral libraries (assuming greater than 85 % similarity). Wherever possible, authentic standards were used to verify the retention times and mass spectra of the compounds. Quantitative analysis was performed by area percentage (integrated and normalized).

### 2.6 Biological assays

#### 2.6.1 Bacterial strains and antimicrobial testing

Dra. Maria Aparecida Vasconcelos Paiva Brito from “Embrapa Gado de Leite” (Juiz de Fora, Minas Gerais, Brazil) kindly provided the five strains isolated from subclinical mastitis patients. The antimicrobial activity of the aqueous phase of *T*. *catappa* was tested against the following bacterial strains isolated from subclinical mastitis infections: *S. aureus* 5455, coagulase-negative staphylococci 5430, *P. aeruginosa* 4511*, Klebsiella pneumoniae (K. pneumoniae)* 7313*, and E. coli* 3830. The microorganism cultures were maintained in Mueller Hinton Broth (Himedia) at 4 °C until use. Prior to antimicrobial tests, the microorganisms were cultured at 37 °C for 24 h.

For the agar diffusion method, adapted from Valgas and coworkers [61], a direct suspension was used, where bacterial colonies were inoculated directly in 0.85 % saline until a turbidity corresponding to 0.5 of the McFarland scale was reached. This means that there are approximately 1.5 x 10^8^ colony-forming units (CFU)/mL.

We used an aliquot of 0.3 mL and seeded it on the surface of the Muller Hinton agar medium in Petri dishes, which were incubated at 37 °C for 30 min. Five microliters of the pyrolysis fractions (TAF1, TAF2, TAF3) were applied directly to the agar medium, making a hole in the surface; then, the plates were incubated at 37 °C for 24 h. Sensitivity was determined by measuring the diameter of the growth inhibition halo, and the test was repeated at least three times.

The minimal inhibitory concentration (MIC) was determined. First, the three fraction dilutions (1000–1 μg/mL) were prepared in a 96-well microplate, with a volume of 100 mL in each well. Second, 100 mL of bacterial inoculum containing 10^8^ CFU/mL was added. Subsequently, the plates were incubated in a bacteriological oven at 37 °C for 16–20 h. In this experiment, pure culture medium with the bacterial inoculum was used as a negative control, and the drugs for clinical use, vancomycin for gram-positive bacteria and gram-negative ciprofloxacin, were used as positive controls.

After the incubation period, 20 μL of resazurin (0.01 % 7-hydroxy-3H-phenoxazine-3-one-10-oxide-SIGMA) was diluted in sterile distilled water and reincubated for another 1 h. The MIC values were determined by visual reading after revelation with resazurin, a colorimetric indicator of oxidereduction that facilitates the verification of bacterial growth and metabolism measurements. The blue color indicates absence, and the pink color indicates the presence of viable cells during growth. The MIC is defined as the lowest concentration of an antimicrobial agent capable of completely inhibiting visible bacterial growth in vitro [62]. The experiments were performed in triplicate.

The minimum bactericidal concentration (CMB) was characterized as the concentration of the drug capable of inhibiting 99.9 % of bacterial growth. The assay adapted from Perumal and Mahmud [63] was performed after MIC determination by pealing on BHI medium plates the contents of the wells in which the MIC and higher concentration were measured. Three duplicate replicates were performed to validate the results.

### 2.7 Mammalian cell culture

The B16F10 murine melanoma cell line from a C57BL/6J mouse was cultured in Dulbecco's modified Eagle's medium (DMEM) supplemented with 10 % inactivated fetal bovine serum (FBS) along with 1 % antibiotics (10,000 units/mL penicillin and 10 mg/mL streptomycin). Peritoneal macrophages were collected by washing Swiss Webster mice aged 4–6 weeks with phosphate-buffered saline (PBS, 1X) via peritoneal injection. After lavage, the samples were centrifuged at 400×*g* for 10 min. The supernatant was discarded, and the pellet was resuspended in Roswell Park Memorial Institute (RPMI) 1640 medium supplemented with 10 % inactivated fetal bovine serum (FBS) and 1 % antibiotics (10,000 units/mL penicillin and 10 mg/mL streptomycin). The macrophages were plated in 96-well microplates for 40 min for adhesion and incubated at 37 °C in 5 % CO_2_ in a humidified atmosphere. After 40 min, the cells were washed and resuspended in RPMI for at least 24 h at 37 °C in 5 % CO_2_ in a humidified atmosphere before any experiments.

#### 2.7.1 Lactate dehydrogenase (LDH)

The LDH assay evaluates necrosis-induced cell death. Cells that have suffered great damage and lose the integrity of their membranes release the enzyme lactate dehydrogenase into the extracellular medium. Thus, this enzyme was identified in the extracellular medium, and its presence is proportional to the extent of plasma membrane damage [[63], [64], [65]]. Cells were grown in 96-well plates at 37 °C in a humidified atmosphere with 5 % CO2. The culture was treated with 500 μg/mL, 250 μg/mL, 125 μg/mL and 31.2 μg/mL substances for 24 h. In the LDH assay, the working reagent was prepared according to the specifications of Bioclin (LDH UV Kit) at the time of reading and was added to the wells. LDH activity in the extracellular medium was measured at 3 min by measuring the absorbance at 340 nm with a SpectraMax M5 spectrophotometer. Cells not treated with the compounds were considered the negative control, and cells treated with the surfactant Triton-X 100 at a 0.5 % concentration were considered the positive control for cell death.

### 2.8 Statistical analysis

The data are expressed as the mean ± standard error of the mean (SEM). Groups were compared using one-way ANOVA. A p value less than 0.05 was considered significant. Statistical analysis was performed using GraphPad Prism 6.0 software (GraphPad Software Inc., San Diego, USA).

## 3 Results and discussion

### 3.1 Pyrolysis process

The moisture content of the fruit obtained by the difference between the initial and final masses of the samples was approximately 70 %. Therefore, the drying step before the process is essential, as its moisture percentage is high and can interfere with the pyrolysis results.

We obtained four fractions in the process based on mass percentage, with average yields of bio-oil (5.52 ± 0.4 %), biochar (37.55 ± 2.8 %), gas (20.71 ± 2.0 %) and aqueous phase (36.22 ± 2.0 %). Biochar and bio-oil are usually the most exploited for their chemical composition, physical-chemical proprieties, and applications. The gas is evaluated for return to the process as fuel in the heating of the pyrolysis furnace.

The aqueous fraction had an acidic pH, brown color, and strong odor. Several substances have previously been identified in aqueous fractions of pyrolysis, in which phenolic compounds and carboxylic acids predominate [36,60]. However, these compositions are mainly dependent on the biomass and the experimental conditions of pyrolysis.

### 3.2 Gas chromatography analysis of the aqueous fractions

Liquid‒liquid extractions were used to isolate the organic compounds dissolved in water for subsequent analysis by GC‒MS. The identified compounds for which the peak similarity was greater than 85 % were obtained from NIST in the hexane, dichloromethane, and ethyl acetate fractions (Table 1).

**Table 1:** Compounds identified in the hexane, dichloromethane and ethyl acetate fractions obtained from the liquid‒liquid extractions of the aqueous fraction.

Compounds	Relative content (%)
**Phenolic compounds**	
Phenol	1.37
1,2-benzenediol *(pyrocathecol)*	12.48
1,2,3-benzenetriol *(pyrogallol)*	1.29
2-methylphenol	0.41
2,4-dimethylphenol	0.26
2,6-dimethylphenol	0.17
2,4,6-trimethylphenol	0.30
3-methyl-benzene-1,2-diol	0.68
2-methyl-benzene-1,4-diol	0.84
3-methyl-benzene-1,4-diol	5.04
4,5-dimethyl-benzene-1,3-diol	0.41
2-methoxyphenol *(guaiacol)*	5.08
4-methoxyphenol *(mequinol)*	9.13
2,6-dimethoxyphenol *(syringol)*	10.01
2,4-dimethoxyphenol	0.16
3,5-dimethoxyphenol	0.36
4-methyl-2-methoxyphenol *(4-methylguaiacol)*	10.91
3-methyl-2-methoxyphenol	0.42
3-methoxy-benzene-1,2-diol	1.71
4-ethyl-2-methoxyphenol *(4-ethylguaiacol)*	3.39
2-methoxy-4-propylphenol *(4-propylguaiacol)*	0.39
1-(4-hydroxy-3-methoxyphenyl)-propanone *(guaiacylacetone)*	2.66
1-(4-hydroxy-3-methoxyphenyl)-ethanone*(acetoguaiacone)*	0.68
4-(1-hydroxy-ethyl)-2-methoxyphenol	0.37
4-(2-propenyl)-2-methoxyphenol *(eugenol or allylguaiacol)*	0.33
4-(1-propenyl)-2-methoxyphenol *(isoeugenol)*	0.36
6-methoxy-eugenol	0.29
4-methoxy-3-(methoxymethyl)-phenol	1.70
4′-hydroxy-3′,5′-dimethoxyacetophenone (acetosyringone)	0.34
***∑ phenolic compounds***	71.54
**Furan compounds**	
Furfural	10.08
5-methylfurfural	2.38
5-hydroxy-methylfurfural	2.09
2-furanmethanol	2.04
2-furilmethylketone	2.33
3-methyl-fura-2,5-dione	0.21
2-(5H)-furanone	0.30
tetrahydro-2-furanmethanol	0.07
2-ethoxytetrahydrofuran	0.03
2-(methoxymethyl)-furan	0.13
2-furylmethylacetate	0.23
2-tetrahydrofurylmethyl	0.31
***∑****Furan compounds*	21.20
**Ketones**	
1-hydroxy-pentan-2-one	0.36
pentan-2-one	0.09
2-methyl-cyclopent-2-en-1-one	1.20
3-methyl-cyclopent-2-en-1-one	0.17
2,3-dimethyl-cyclopent-2-en-1-one	0.26
3-methyl-cyclopenta-1,2-dione	1.15
***∑****ketones*	3.23
**Nitrogenous compounds**	
methylpyrazine	0.34
2-hydroxypropanenitrile	0.17
4,5-dimethyl-1-H-imidazole	0.45
***∑****nitrogenous compounds*	0.96
**Others compounds (aromatic hydrocarbons, alcohols, ethers, aldehydes, acids)**	
1,2,3- trimethoxy-5-methylbenzene	0.45
2-ethoxypropane	0,26
4-ethoxy-3-anisaldehyde	0.32
4-hidroxy-3,5-dimethoxy-benzaldehyde	0.34
Ethylbenzene	0.13
*p*-xylene	0.09
m-xylene	0.24
butane-1,3-diol	0.12
4-hydroxy-butanoic acid	0.50
Propylene glycol	0.12
2-methyl-pent-2-enal	0.24
acetic acid	0.14
butanoic acid	0.12
***∑***	3.07

The chemical composition of the fruit is complex, and analysis of the chemical composition of the aqueous pyrolysis fraction revealed phenolic (71.54 %) and furan (21.20 %) compounds, which are soluble in water via interactions via hydrogen bonds.

Phenolic compounds are formed mainly due to the decomposition of lignin and tannins. Lignin is a highly branched three-dimensional structure with cross-linked layers of hydroxyl- and methoxy-substituted phenylpropane units [66]. The main phenolic compounds derived from lignin pyrolysis are syringol, guaiacol (methoxy phenols) and catechols [59].

Tannins are naturally occurring polyphenolic compounds and are categorized according to their chemical structure into hydrolysable tannins (gallotannins, derivatives of gallic acid (3,4,5-trihydroxylbenzoic acid) and ellagitannin) and condensed (or proanthocyanidins) polymers of flavan-3-ols (cathechin) and/or flavan-3,4-diols or a mixture of the two [67]. Pyrocatechol and pyrogallol are the main thermal decomposition products of condensed tannins [68].

The presence of aromatic hydrocarbons such as methyl benzene and nitrogen-containing compounds can be attributed to the thermal decomposition of proteins [69]. Acetic acid and butanoic acid are derived from the degradation of hemicelluloses [59].

Phenols with methyl substituents and ketones, such as substituted cyclopentenones, are the main constituents of the pyrolysis of xylan (hemicelluloses) [56]. Furan compounds, C3–C5 ketones, aldehydes, alcohols and substituted cyclopentenones are derived from cellulose pyrolysis.

**Fig. 2:**
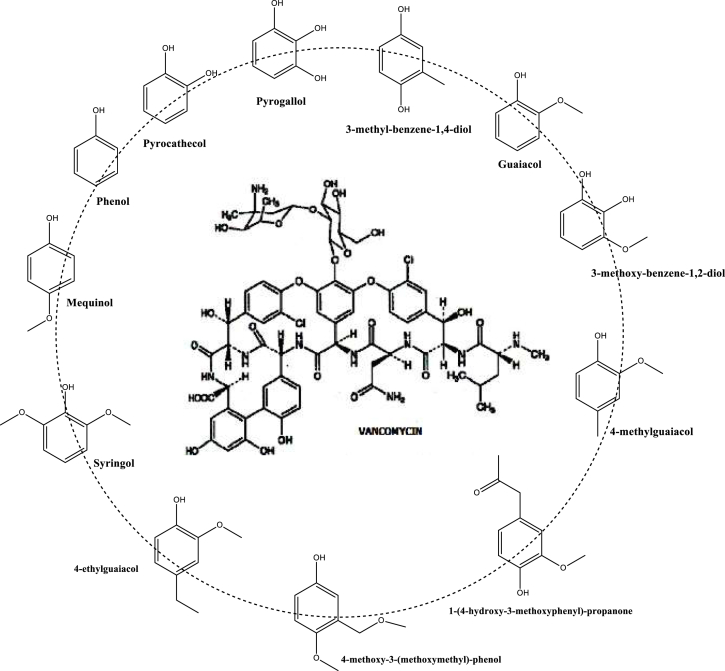
Correlations between phenolic compounds found in pyrolysis water and the phenolic groups of vancomycin.

As the chemical composition of the aqueous fraction contains several phenolic and furanic compounds, which are broadly described as having a range of pharmacological activities, the same was used for tests with *S. aureus, K. pneumoniae, P. aeruginosa* and *E. coli* in bovine mastitis. The two substances used as reference standards were ciprofloxacin and vancomycin, which present phenolic groups in their structure, as well as the aqueous fraction of *T*. *catappa* pyrolysis, which is composed mainly of phenolic compounds (Fig. 2).

### 3.3 Correlation of the composition of the aqueous fraction and activity of bovine mastitis bacterial strains

**Fig. 3:**
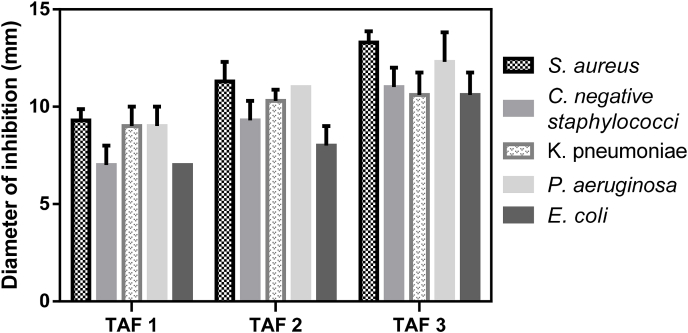
Diameter of inhibition from Terminalia fractions. **TAF1**= Terminalia aqueous fraction one; **TAF2**= Terminalia aqueous fraction two; TAF3= Terminalia aqueous fraction three. These data were obtained in triplicate on at least three distinct days.

The results from the agar diffusion method (Table 2) show antibacterial activity in all pyrolysis aqueous fractions of *T. catappa* tested against gram-positive strains such as gram-negative strains. Moreover, we observed an increase in activity in the last fraction, where the active molecules may be at a higher concentration. Additionally, wood vinegar obtained from *Litchi chinensis* showed antibacterial activity, causing a 19 mm inhibition zone [70]. Other studies have demonstrated inhibition halo formation of up to 33 mm and 29.5 mm in aqueous fractions obtained from *Eucalyptus urograndis and Mimos atenuiflora* plants, respectively [71]. Fractions 2 and 3 were mainly active against the *S. aureus* and *P. aeruginosa* strains (Fig. 3). The inhibition diameters of 90 % ethanolic extracts from *T. catappa* ranged from 9.06 mm (*S. aureus*) to 6.5 mm (*P. aeruginosa*) [72]. TAF3 reached values greater than 10 mm for all tested bacteria.

**Table 2:** Antimicrobial susceptibility test (TSA) and the agar diffusion method were performed with pyrolysis aqueous fractions of *T*. *catappa.* C+^b^ -Positive controls: vancomycin (a gram-positive strain) and ciprofloxacin (a gram-negative strain)**. TAF 1:** Terminalia aqueous fraction one; **TAF 2:** Terminalia aqueous fraction two; **TAF 3:** Terminalia aqueous fraction three. Five microliters of the pyrolysis fractions (TAF1, TAF2, TAF3) were applied directly to the agar media. aReferent results of 3 different tests.

Inhibition zone (mm)^a^				
Strains	TAF 1	TAF 2	TAF 3	C+^b^
*S. aureus*	9.3 ± 0.57	11.3 ± 1	13.3 ± 0.57	15 ± 0
Coagulase-negative staphylococci	7 ± 1	9.3 ± 1	11 ± 1	16 ± 0
*K. pneumoniae*	9 ± 1	10.3 ± 0.57	10.6 ± 1.15	29.6 ± 1.54
*P. aeruginosa*	9 ± 1	11 ± 0	12.3 ± 1.52	30 ± 0
*E. coli*	7 ± 0	8 ± 1	10.6 ± 1.15	28.3 ± 1.54

a Results of 3 different tests.

b C + - Positive control: vancomycin (a gram-positive strain) and ciprofloxacin (a gram-negative strain). **TAF 1:** Terminalia aqueous fraction one; **TAF 2:** Terminalia aqueous fraction two; **TAF 3:** Terminalia aqueous fraction three.

After the observation of Terminalia activity, the MIC test revealed a lower concentration capable of inhibiting bacterial growth. The MICs of the first aqueous fraction of *Terminalia* (TAF 1) were 1000 μg/mL and 500 μg/mL, whereas the MIC of the second aqueous fraction was only 250 μg/mL, and the MICs of the third aqueous fraction were 250 μg/mL and 125 μg/mL (Table 3). Using pyroligneous palm shells, Ariffin and collaborators [73] demonstrated growth inhibition with MICs between 1.95 and 3.91 mg/mL for both gram-positive and gram-negative strains. Recent findings point to MIC values for both water-soluble phases of the pyrolytic process of *Eucalyptus* spp. being the same for *S. aureus* and *E. coli* bacteria at 2.10 and 2.17 g/L, respectively [74]. In contrast, the aqueous pyrolytic fraction of *T. catappa* presented lower MIC values than did previous findings for inhibiting gram-positive and gram-negative bacteria.

**Table 3:** Minimal inhibitory concentration (MIC) of pyrolysis aqueous fractions. **TAF1:** Terminalia aqueous fraction one; **TAF2:** Terminalia aqueous fraction two**; TAF3:** Terminalia aqueous fraction three.

MIC (μg/mL)			
Strains	TAF 1	TAF 2	TAF 3
*S. aureus*	500	250	125
Coagulase-negative staphylococci	500	250	125
*K. pneumoniae*	500	250	250
*P. aeruginosa*	500	250	125
*E. coli*	1000	250	250

**TAF1:** Terminalia aqueous fraction one; **TAF2:** Terminalia aqueous fraction two; **TAF3:** Terminalia aqueous fraction three.

The bactericidal concentration capable of killing 99.9 % of the bacteria varied between 1000 and 250 μg/mL, with the lowest concentration being found mostly in the third aqueous fraction (Table 4). These values were found to be close to those of Patra and coauthors [75] in the assay performed with the oily fraction of the *Pinus densiflora* plant, and the bactericidal concentration ranged from 1000 μg/mL to 500 μg/mL for *Escherichia coli* and *Samolnella tiphymurium*.

**Table 4:** Minimum bactericidal concentration (MBC) of the pyrolysis aqueous fractions. **TAF1:** Terminalia aqueous fraction one; **TAF2:** Terminalia aqueous fraction two**; TAF3:** Terminalia aqueous fraction three.

MBC (μg/mL)			
Strains	TAF 1	TAF 2	TAF 3
*S. aureus*	500	500	250
Coagulase-negative staphilococci	1000	250	250
*K. pneumoniae*	1000	500	500
*P. aeruginosa*	500	250	250
*E. coli*	1000	500	500

**TAF1:** Terminalia aqueous fraction one; **TAF2:** Terminalia aqueous fraction two; **TAF3:** Terminalia aqueous fraction three.

Previous studies have shown that the bactericidal activity of pyrolytic derivatives is related to the large presence of phenolic compounds [76]. Our phytochemical findings revealed a high presence of phenolic compounds in the aqueous fraction (71.54 %). These data may be a good indication of the antimicrobial activity in our findings, since structures of this chemical class are present in conventional medicines such as the aforementioned vancomycin (Fig. 2). Interestingly, Kocaçalişkan and colleagues [77] showed that the phenolic derivatives catechol and pyrogallol had antibacterial effects on all bacteria at concentrations of 5 and 10 mM. In this study, we observed a significant presence of catechol in the analysis (12.48 %) and a lower presence of pyrogallol (1.29 %). Previous data indicate the ability of catechol to adhere to bacteria, which leads to membrane rupture due to inhibition of the enoyl-acyl transport enzyme protein reductase (FabI), impairing the synthesis of fatty acids via a mechanism similar to that of the phenolic derivative drug triclosan [78,79]. Furthermore, pyrogallol has been shown to reduce the growth of methicillin-resistant *Staphylococcus aureus* by targeting cell membrane fatty acids, proteins/peptides, polysaccharides/carbohydrates, and cell wall peptidoglycan [80]. Together, these data may point to the mechanism of action of the extract and the importance of phenolic compounds in bacterial inhibition.

Another chemical group identified in the extract was furanic compounds (21.20 %). Furfural, the largest compound in the class (10.08 %), has already demonstrated promising inhibitory activity against the bacteria *Bacillus subtilis* and Salmonella, with MICs of 27 and 29 nM, respectively [81]. Interestingly, according to the concentration of furfural used, changes in DNA, including deletions, were observed in *E. coli,* and mutagenic effects were observed in the TA100 strain of *Salmonella typhimurium* [82,83]. Therefore, the presence of furfural suggests the antimicrobial potential of the extract when it is added to phenols.

### 3.4 Cytotoxicity in mammalian cells

**Fig. 4:**
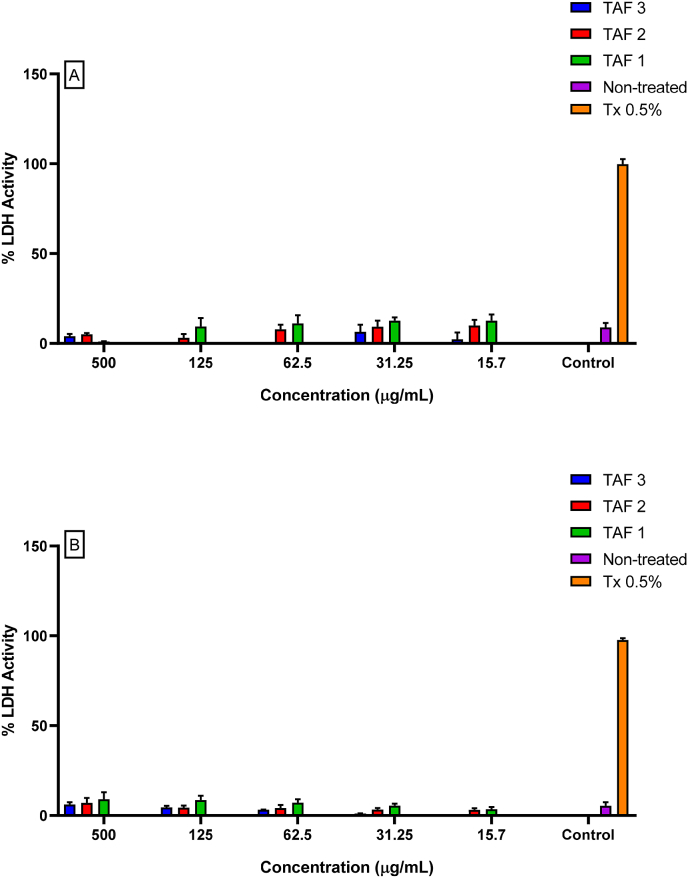
LDH release in the extracellular medium of B16F10 cells after 24 h of pyrolysis at 500 μg/mL (TAF only), 250 μg/mL, 125 μg/mL and 31.2 μg/mL (TAF1, TAF2 and TAF3). The results are representative of at least three experiments performed on distinct days in triplicate. ****p < 0.001 compared to untreated cells.

The fact that a substance affects the metabolism of cells does not necessarily reflect cell death. Primarily, the necrosis process is the most harmful to the body's homeostasis. Therefore, the LDH assay was performed with the objective of evaluating whether the substances were toxic to mammalian cells. We evaluated a mouse primary cell type, macrophages, and a mouse epithelial cell line, B16F10 cells. When we performed the LDH assay on B16F10 cells (Fig. 4A) or macrophages (Fig. 4B), we did not observe a significant difference between untreated cells and cells treated with the pyrolysis fractions of Terminalia. Therefore, none of the substances led to necrotic cell death.

LDH in the extracellular medium was less than 20 % less than that in untreated cells. Therefore, the pyrolysis fraction caused only a basal release of this enzyme. Shivananjappa and Joshi [84] treated cells with 15.7, 31.25, 62.5, 125 and 500 μg/mL *Terminalia arjuna*, which belongs to the same genus as *T*. *catappa*, for 24 h. The treatment did not alter LDH leakage into the culture medium, indicating no cytotoxic effect. However, concentrations higher than 250 μg/mL caused discrete LDH release at all concentrations in both cell types. The aqueous extract of *T. cattapa* leaves prevented gene mutations and suppressed intracellular free radicals involved in bleomycin-induced genotoxicity in CHO cells. These effects have been attributed to phenols, such as tannins [85,86].

Interestingly, an in vivo experiment developed from the crude aqueous extract of *T. catappa* evaluated its toxic effect on Sprague‒Dawley rats over 14 days based on its lethal toxicity effect and nutritional parameters. The administration of 0.5 g/kg, 1.0 g/kg or 3.0 g/kg sample did not cause lethality or physiological alterations in the analyzed parameters during the study period [87]. Our results showed that the tested concentrations of different pyrolic fractions of *T. catappa* did not cause toxicity to cells from the first barrier (epithelial cells) or defense system (macrophages). Consequently, Terminalia's pyrolysis fractions are not toxic, but as they may affect the metabolism of mammalian cells, other forms of death over time, such as apoptosis, cannot be ruled out. Taken together, our data confirm the good tolerability of this plant that has already been addressed in previous studies.

## 4 Conclusions

Pyrolysis is an essential tool in the proper disposal of MSW waste. In this study, the slow pyrolysis process was applied in the treatment of *T. catappa* fruits, generating a high yield of the liquid fraction (41.74 %), which can be easily separated during the process into aqueous and organic phases. GC‒MS analyses indicate that phenols are the main components, followed by furanic compounds. The aqueous fraction was studied for its ability to control bovine mastitis and showed important activity for the control of several gram-positive and gram-negative bacteria related to bovine mastitis. Moreover, LDH released into the extracellular medium of B16F10 cells and mouse peritoneal macrophages demonstrated low toxicity. The antimicrobial activity of pyroligneous acid has been attributed to the presence of compounds such as phenols. The presence of pyrocatechol and pyrogallol, along with other phenols and furfural, may explain the antibacterial activity of this fraction. animal infections.

## CRediT authorship contribution statement

**Rafaelle Vinturelle:** Writing – original draft, Methodology, Investigation, Formal analysis. **Taissa da Silva Cabral:** Investigation, Formal analysis. **Pamella C.O. de Oliveira:** Methodology, Investigation. **Juliana P. Salles:** Methodology, Investigation. **Juliana V. Faria:** Methodology, Investigation. **Guilherme P. Teixeira:** Writing – original draft, Supervision. **Robson X. Faria:** Writing – original draft, Validation, Project administration, Methodology, Funding acquisition, Formal analysis, Conceptualization. **Márcia C.C. Veloso:** Writing – original draft, Visualization, Methodology, Conceptualization. **Gilberto A. Romeiro:** Visualization, Supervision, Investigation, Conceptualization. **EvelizeFolly das Chagas:** Resources, Project administration, Methodology, Investigation, Formal analysis, Conceptualization.

## Declaration of competing Interest

The authors declare that they have no known competing financial interests or personal relationships that could have appeared to influence the work reported in this paper.

## Data Availability

Data will be made available on request.
